# Sequencing and Characterization of αs2-Casein Gene (*CSN1S2*) in the Old-World Camels Have Proven Genetic Variations Useful for the Understanding of Species Diversification

**DOI:** 10.3390/ani13172805

**Published:** 2023-09-04

**Authors:** Alfredo Pauciullo, Carmine Versace, Giustino Gaspa, Neyrouz Letaief, Sonia Bedhiaf-Romdhani, Andrea Fulgione, Gianfranco Cosenza

**Affiliations:** 1 Department of Agriculture, Forest and Food Sciences, University of Torino, 10095 Grugliasco, Italy; 2 Laboratory of Animal and Forage Production, National Agricultural Research Institute of Tunisia, University of Carthage, Ariana 1004, Tunisia; 3 Department of Agriculture, University of Napoli Federico II, 80055 Portici, Italy

**Keywords:** *CSN1S2*, αs2-casein, dromedary, Bactrian camel

## Abstract

**Simple Summary:**

Milk proteins are crucial for a healthy diet and offer various health benefits. Genetic variants of milk proteins are often drivers of different technological and nutritional milk characteristics, as shown in cow milk research. Similarly, genetic variants have been identified in camel caseins, but information on the αs2-casein gene (*CSN1S2*) is limited. Therefore, this study investigated the *CSN1S2* gene in Old-World camels (*Camelus bactrianus* and *Camelus dromedarius*). Both camel species share most of the gene characteristics, including the presence of the exon 12, formerly not described in large camels. Two novel allelic variants were discovered: one was a missense mutation (Bactrian camel), and the other was a noncoding mutation (dromedary camel). The gene promoter mutations affected the binding sites of transcription factors, and there were differences in microRNA seed sequences due to a single nucleotide polymorphism (SNP) at the 3′Untranslated region (UTR). The gene structure and interspersed element locations indicate a divergence between *Tylopoda* and *Ruminantia*.

**Abstract:**

The *CSN1S2* gene encodes αs2-casein, the third most abundant protein in camel milk. Despite its importance in foals, human nutrition, and dairy processing, the *CSN1S2* gene in camels has received little attention. This study presents the first complete characterization of the *CSN1S2* gene sequence in Old-World camels (*Camelus bactrianus* and *Camelus dromedarius*). Additionally, the gene promoter, consisting of 752 bp upstream of exon 1, was analyzed. The entire gene comprises 17 exons, ranging in length from 24 bp (exons 4, 8, 11, and 13) to 280 bp (exon 17). Interesting was the identification of the exon 12 in both species. The promoter analysis revealed 24 putative binding sites in the Bactrian camel and 22 in dromedary camel. Most of these sites were typical elements associated with milk protein, such as C/EBP-α, C/EBP-β, Oct-1, and AP1. The SNP discovery showed relatively high genetic diversity compared to other camel casein genes (*CSN1S1, CSN2,* and *CSN3*), with a total of 34 polymorphic sites across the two species. Particularly noteworthy is the transition g.311G>A in the *CSN1S2* promoter, creating a new putative consensus binding site for a C/EBP-β in the Bactrian camel. At the exon level, two novel variants were found. One was detected in exon 6 of the Bactrian camel (g.3639C>G), resulting in an amino acid replacement, p.36Ile>Met. The second variant was found in noncoding exon 17 of dromedary *CSN1S2* (g.1511G>T). Although this mutation occurs in the 3′-UnTranslated Region, it represents the first example of exonic polymorphism in the *CSN1S2* for this species. This SNP also affects the binding sites of different microRNAs, including the seed sequence of the miRNA 4662a-3p, highlighting its role as a regulatory factor for *CSN1S2* gene. A PCR-RFLP was set up for genotyping a dromedary Tunisian population (*n* = 157), and the minor allele frequency was found to be 0.27 for the G allele, indicating a potential yield improvement margin. The interspersed elements (INEs) analysis revealed 10 INEs covering 7.34% and 8.14% of the *CSN1S2* sequence in the Bactrian and dromedary camels, respectively. Furthermore, six elements (A, B, F, H, I, and L) are shared among cattle and camels and are partially found in other ruminants, suggesting a common ancestral origin of these retrotransposons. Conversely, elements C, D, E, and G are specific to camels.

## 1. Introduction

The αs2 casein (CN) is a key phosphoprotein that is present in ruminants’ milk, alongside other caseins (αs1, β, and k) [1]. The *CSN1S2* gene encodes this protein [2]. While this gene is mapped on chromosome 6 in cattle and goats [2], it is located on chromosome 2 in camels [3]. The content of αs2-CN in milk varies greatly among species. For example, while absent in human milk [4], it constitutes approximately 9.5% of the total casein content in camel milk [5] and 10% in cow milk [6].

Studies of αs2-CN have revealed diverse protein and DNA polymorphisms across different species. In small domestic ruminants (goats and sheep), researchers have identified at least eight *CSN1S2* alleles associated with three different αs2-CN content levels [7,8,9,10,11]. Among larger ruminants, cattle contain four variants (A, B, C, and D) [12], and the buffalo has eight alleles (A, B, B1, B2, C, D, E, and F) [13]. 

Within the *Camelidae* family, αs2-CN gene (*CSN1S2*) studies have ranged from the molecular characterization of the cDNA in llamas (*Lama glama*) [14] to a global analysis of the casein gene cluster in major camelids [3] to a report by Ryskaliyeva et al. [15] on alternative splicing events for the camel αs2-casein. More recently, two variants have been described in a United Arab Emirates dromedary camel population [16]—one (c.-19A>C) located in the promoter region that may affect gene expression, and one (c.403-9_403-4delTTTTCT) that affects a splice site.

The importance of camel αs2-CN to foals, human nutrition, and dairy processing warrants a greater understanding of camel *CSN1S2* diversity. The demand for a comprehensive analysis of *CSN1S2* in Old-World camels also comes from a growing international interest in the health benefits and variety of foods produced from camel milk [17]. To this end, a study was developed with two main goals. The first goal was to sequence, thoroughly annotate, and compare the entire *CSN1S2* gene and its regulatory regions in Bactrian (*Camelus bactrianus*) and dromedary (*Camelus dromedarius*) camels. The second goal was to explore the genetic diversity in these species and identify markers that may be useful for selective breeding. Particular attention was paid to the SNP g.15110G>T for the potential effect on miRNA binding sites.

## 2. Materials and Methods

### 2.1. DNA Samples

The samples used in this study were obtained from previous studies and are part of collections of the University of Torino (Northern Italy) and the Justus-Liebig University of Giessen (Germany). Specifically, 157 DNAs were originally isolated from individual blood samples of Tunisian dromedaries (*C. dromedarius*), reared in four different regions of the country, representing different ecotypes. All procedures were conducted under strict adherence to ethical treatment and animal welfare national legislation [18]. Additionally, 10 individual DNAs from Bactrian camels (*C. bactrianus*) were collected from Wilhelma Zoo (Stuttgart, Germany) [19,20].

Genomic DNA was extracted using the standard phenol–chloroform method as described by Sambrook and Russell [21]. The concentration of DNA and the OD_260/280_ ratio were determined using a NanoDrop ND-1000 Spectrophotometer (Thermo-Scientific, Waltham, MA, USA).

### 2.2. PCR Amplification Conditions and Sequencing

For the whole camel *CSN1S2* gene sequencing, we selected 30 test samples (20 dromedaries randomly chosen from each region and 10 Bactrians). For amplification and sequencing, we relied on the dromedary genome (GenBank ID: NC_044512 region 69170091..69184631) as a template and DNAasis-Max ver. 3.0 software (Hitachi) to design 30 different primers (Supplementary Table S1). All primers were initially tested by gradient PCR in a final volume of 15 µL to determine the optimal annealing temperature. The PCR reaction mix included 50 ng of genomic DNA, 1× PCR buffer (Promega), 2.5 mM of MgCl_2_, 200 µg dNTP, 1 pmol of each primer, and 0.75 U of *GoTaq^®^ Flexi* DNA Polymerase (Promega). The thermal profiles consisted of an initial denaturation at 95 °C (3 min), followed by 35 cycles at 95 °C (45 s), with annealing temperatures and times specific to each amplicon, then 72 °C (2 min), and ending with final extension at 72 °C (5 min), using a T100 thermal cycler (Bio-Rad). After PCR, the products were purified using NucleoSpin Gel and PCR cleanup (Machery-Nagel), and sequencing was outsourced to Eurofins Genomics (Ebersberg, Germany), using TubeSeq Supreme service.

### 2.3. Genotyping of Dromedaries by TaqI PCR-RFLP

To genotype the Tunisian dromedary population for the SNP g.15110G>T, we used the PCR-RLFP method. The digestion was performed using *TaqI* FastDigest endonuclease (5′….AAATCN↓….3′) at 65 °C for 15 min per the manufacturer’s guidelines (Thermofisher Scientific). The digestion products were then analyzed by electrophoresis in a 1.5% agarose gel in 0.5× TBE buffer and stained with ethidium bromide.

### 2.4. Bioinformatics

Gene annotation was performed by the authors using various bioinformatics tools. DNAsis-Max ver. 3.0 software (Hitachi) was used for SNP discovery, homology searches, sequence comparison, and multiple alignments. Interspersed elements (INEs) were identified using RepeatMaskers Web Server (http://repeatmasker.org/cgi-bin/WEBRepeatMasker, accessed on 5 March 2023). For phosphorylation site prediction, signal peptide identification, and mature protein sequence cleavage, we used NetPhos 3.1 (https://services.healthtech.dtu.dk/service.php?NetPhos-3.1, accessed on 8 February 2023) and Signal IP 5.0 (https://services.healthtech.dtu.dk/service.php?SignalP-5.0, accessed on 25 January 2023), respectively. The gene’s promoter underwent putative sequence discovery for transcription factor binding sites using the TFBIND tool (https://tfbind.hgc.jp, accessed on 11 June 2023). For the analysis of the 3′-flanking region for potential microRNA sequences, we turned to the miR database (https://mirdb.org/mirdb/custom.html, accessed on 4 July 2023), and Target Scan (https://www.targetscan.org/vert_80/, accessed on 4 July2023) was used to verify potential target genes.

## 3. Results 

### 3.1. CSN1S2 Gene Structure in Camels

**Figure 1:**
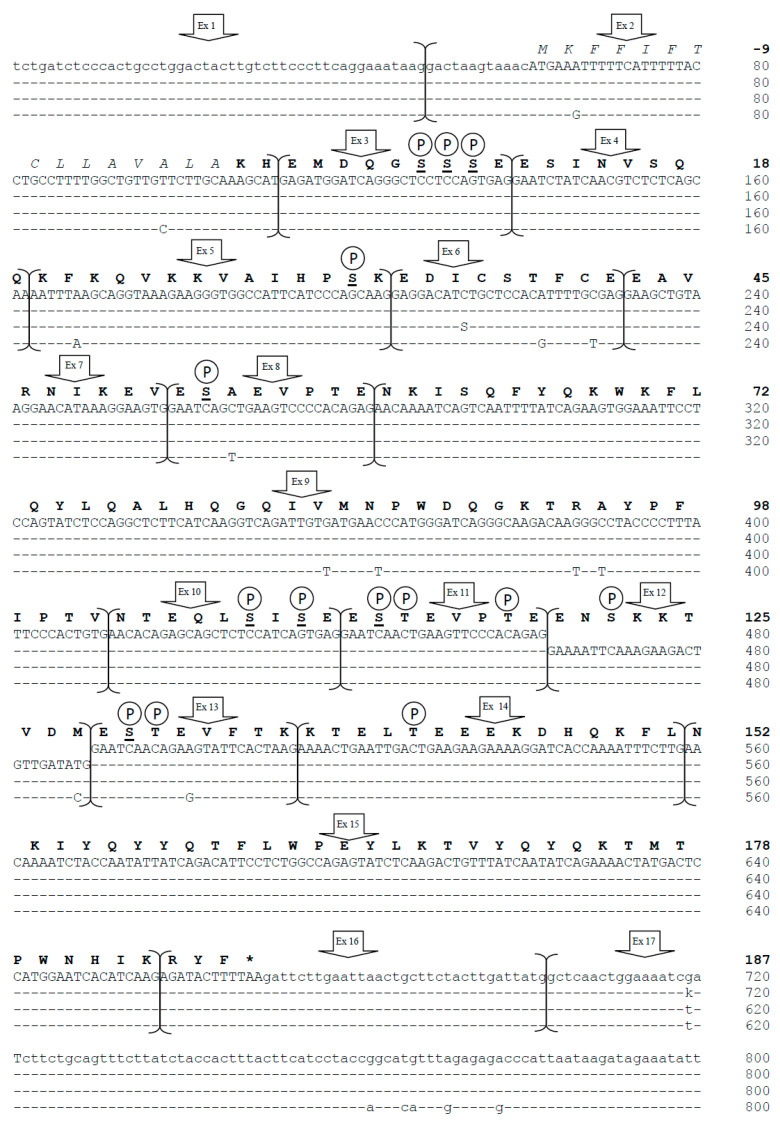

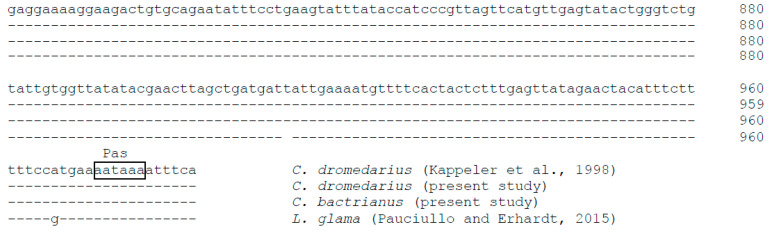
Complete cDNA and exon subdivision of *CSN1S2* sequence (upper line) [5] and comparative alignment with the homologous αs2-casein cDNA of *C. dromedarius* (GenBank ID: OQ730239) and *C. bactrianus* (GenBank ID: OQ730238) of the present study and with that of *L. glama* (EMBL acc. no. LK999989) [14]. Dashes represent identical nucleotides of the upper lines. The 5′- and 3′- Un-Translated Regions (UTR) are in lowercase, and the polyadenylation signal (Pas) is indicated by a box. Within the nucleotide sequence, S = C/G (*C. bactrianus* exon 6) and k = G/T (*C. dromedarius* exon 17). The corresponding mature protein is reported in bold, whereas the signal peptide is in italics, and the asterisk represents the termination stop codon. Putative phosphorylation sites are indicated with P. Phosphorylated serines reported by Kappeler et al. [5] are underlined.

We sequenced the complete *CSN1S2* gene, which encodes αs2-casein in *Camelus bactrianus* and *Camelus dromedarius.* The sequenced regions included 752 bp at the 5′ flanking region and 115/114 bp at the 3′ flanking region and are identified in GenBank as ID numbers OQ730238 (*C. bactrianus*) and OQ730239 (*C. dromedarius*). The entire gene is approximately 15,000 bp long, with 14,735 bp in dromedary and 14,695 bp in Bactrian, consisting of 982 bp in exonic regions and about 14,000 bp in intronic regions (13,753 bp in dromedary and 13,713 bp in Bactrian). Exon 1 (48 bp) and the first 12 bp of exon 2 are noncoding. The signal peptide (15 amino acids) starts translation with the ATG codon of the 13th nucleotide and continues through the following 45 nucleotides of exon 2. The mature peptide (187 amino acids) is encoded by the last 6 nucleotides of exon 2 up to the first 9 bp of exon 16, and the translation stop codon, TAA, is located between the 10th and 12th nucleotides of exon 16. No variations in splice donor and/or acceptor consensus sequences were found. Figure 1 displays the complete cDNA of dromedary and Bactrian camels, including the exon 12 formerly not described in camels, and the encoded protein. The protein analysis revealed 14 putative serine/threonine motifs as phosphorylation sites (Figure 1).

### 3.2. Genetic Variability in Bactrian and Dromedary Camels

Thirty samples (twenty randomly chosen dromedaries and all available Bactrians) were sequenced for SNP discovery. A total of 18 intraspecies polymorphic sites (5 transversions and 13 transitions) were found in Bactrian camels, and 16 intraspecies polymorphisms (6 transversions and 10 transitions) were identified in dromedary camels (Table 1). Comparison of the two species revealed 28 interspecies SNPs (Supplementary Table S2). We also detected a microsatellite (GACA)n that was characterized by 7 repetitions (Bactrians) and 14 (dromedaries) at positions +7173/+7200 and +7182/+7238, respectively.

### 3.3. CSN1S2 Gene Promoter

To explore SNP discovery and identify potential regulatory regions of the αs2-casein gene, we sequenced the 5′-flanking region in both dromedary and Bactrian camels. The analysis of the *CSN1S2* promoter revealed 24 putative binding sites in Bactrian and 22 in dromedary camels (Table 2).

### 3.4. Genotyping of the SNP g.15110G>T by TaqI PCR-RFLP

**Figure 2:**
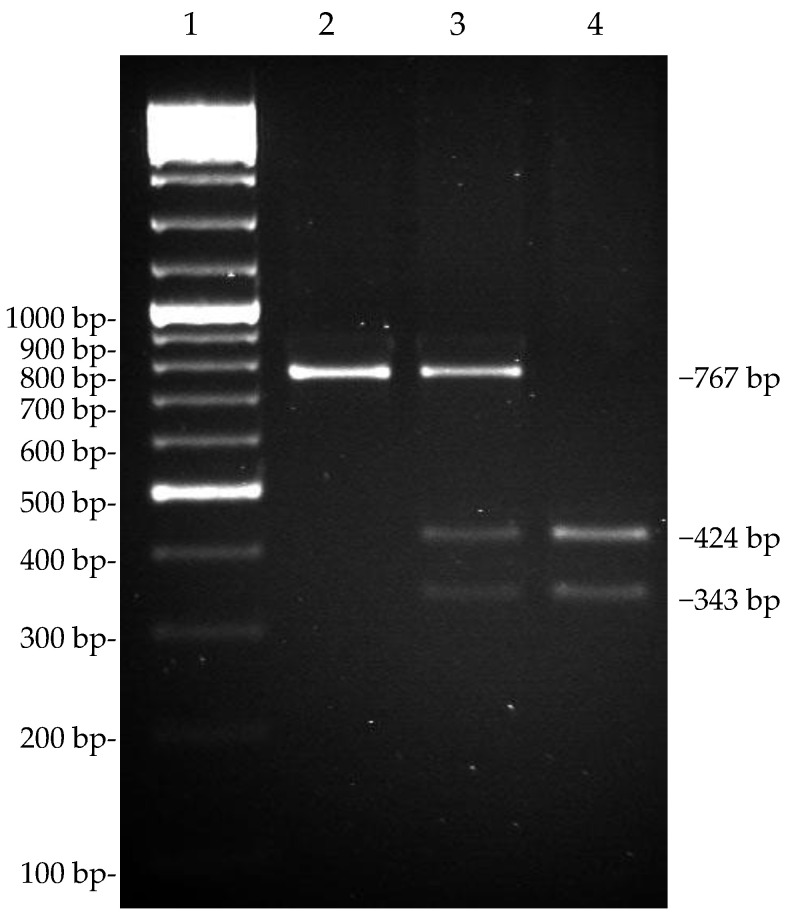
Genotyping of *C. dromedarius CSN1S2* g.15110G>T SNP by Taq I PCR-RFLP. Line 2, TT homozygous samples; line 3, GT heterozygous samples; and line 4, GG homozygous samples. Line 1 is GeneRuler 100 bp DNA Ladder Mix (Thermo Scientific).

We analyzed the SNP (g.15110G>T) located at exon 17 in the 3′ untranslated region (UTR) of dromedary *CSN1S2*, using PCR-RFLP, in a population of 157 animals. The PCR product (767 bp), which was amplified by the primers 5′-GGATAATTAAATGTTCCTTCAAAA-3′ (forward) and 5′-GTGAGAAGTAAAACTGAAGT-3′ (reverse), was digested with endonuclease *TaqI* to identify the alleles. The digestion pattern showed one band (767 bp) for the T/T samples and two bands, 424 bp and 343 bp long, for the G/G samples. The heterozygous samples showed three fragments (Figure 2). The minor allele frequency (MAF) was 0.277 for allele G (Table 3), and the χ^2^ test indicated that the allele frequency did not deviate from the Hardy–Weinberg equilibrium (*p* < 0.05).

### 3.5. Interspersed Elements and microRNA

**Figure 3:**
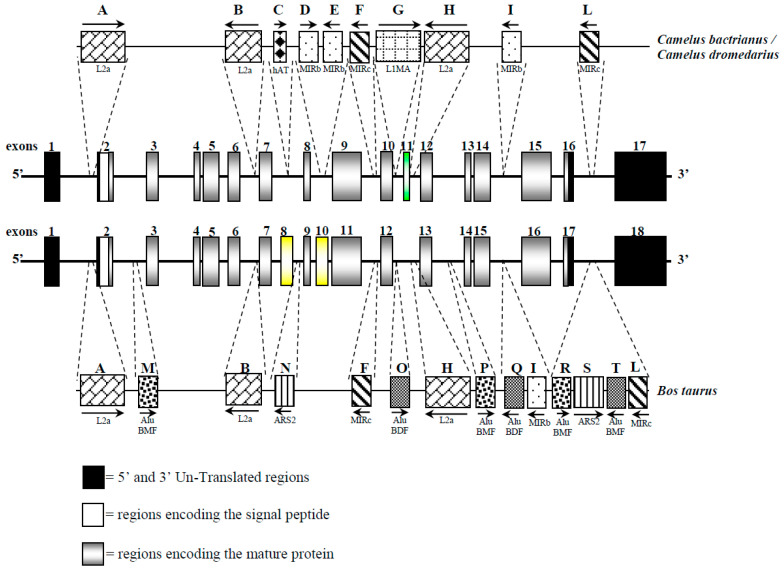
Schematic representation of the *CSN1S2* gene and of the interspersed elements observed in Bactrian/dromedary camel and cattle. Exons 8 and 10 of the cattle *CSN1S2* are indicated as yellow boxes because they have no homologous exons in camels. Exon 11 in camels is indicated as a green box because is a duplication of the exon 8 in the same species. Common interspersed elements are indicated with the same letter.

The bioinformatics analysis allowed us to find and characterize 10 retrotransposons in both camel species’ *CSN1S2* gene (Table 4). To appreciate interspersed elements to drive species diversification, we compared both camel species to the homologous bovine gene (Table 4 and Figure 3). We also identified mature microRNA (miR) sequences affected by the SNP (g.15110G>T) in the 3′-flanking region (Table 5).

## 4. Discussion

The αs2-casein is a major protein found in camel milk, alongside other caseins (αs1, β, and κ), and a complex pattern of whey proteins. In this study, our aim was to characterize the *CSN1S2* gene in Old-World camels, addressing a gap in the literature. Previous research has extensively analyzed the other casein genes, such as *CSN1S1* (αs1-CN), *CSN2* (β-CN), and *CSN3* (κ-CN), with a focus on the discovery of their genetic diversity [19,22,23]. However, information regarding the camel *CSN1S2* gene remained limited. 

We amplified and sequenced the complete *CSN1S2* gene in 30 animals, including 20 randomly chosen dromedaries and 10 Bactrians. The gene structure, consisting of 17 exons and 16 introns, was found to be conserved between the two species. Exon sizes ranged from 24 bp (exons 4, 8, 11, and 13) to 280 bp (exon 17), while intron sizes ranged from 83 bp (intron 4) to 2020/2022 bp (intron 16) in *C. bactrianus* and *C. dromedarius*, respectively. The *CSN1S2* cDNAs showed high homology among the members of the *Tylopoda* family (Figure 1). 

**Figure 4:**
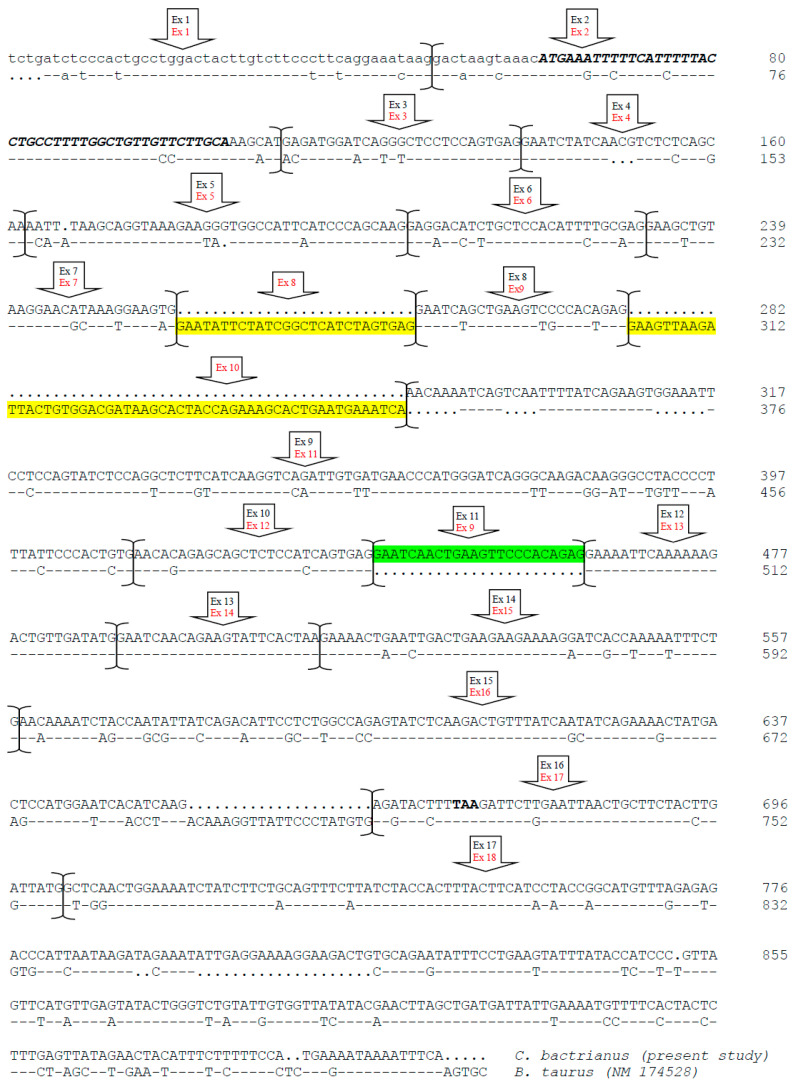
Complete cDNA sequence and exon subdivision of the *CSN1S2* cDNA (αs2-casein) of *C. bactrianus* (present study) and comparative alignment with the homologous bovine cDNA (EMBL acc. no. NM_174528). Dashes represent identical nucleotides of the upper lines, dots are nucleotides that are not present in the comparative analysis, and bold and italics identify nucleotides coding for the signal peptide. The big arrows indicate exons (upper black, the numbering of camel; down red, the numbering of bovine). Exons 8 and 10 of the cattle *CSN1S2* are highlighted in yellow, with no homologous exon in camels. Exon 11 (green highlight) is a duplication of exon 8 in camels.

Of particular interest was the discovery of exon 12, which was present in both Bactrian and dromedary camels, although Kappeler et al. [5] did not describe exon 12 in the cDNA. This exon, 27 bp long, encodes for the peptide ENSKKTVDM and has been identified in llamas cDNA [14]; it was also confirmed by a proteomic approach [15]. Overall, the *CSN1S2* cDNA in camels is 982 bp long, slightly shorter than the cattle counterpart (1028 bp), with an overall sequence similarity of about 62.2% (Figure 4). The comparative analysis of these cDNAs revealed differences, particularly in the number of exons, with camels having 17 exons and cattle having 18. However, the rearrangements are more complex than a simple insertion/deletion. A duplication event was observed in the camel *CSN1S2* cDNA, where exons 8 and 11 shared 22 out of 24 bp. These two exons in camels corresponded to exon 9 in the cattle *CSN1S2* sequence, where no such duplication occurred. On the other hand, the cattle *CSN1S2* had exons 8 and 10, which had no homologous exons in camels; thus, they are considered to be extra exons (Figure 3 and Figure 4).

Phosphorylation sites were analyzed, revealing 14 putative serine/threonine motifs (Figure 1). Phosphorylation is an essential post-translational modification that occurs after caseins synthesis through the action of protein kinases [24]. These kinases phosphorylate serine or threonine residues, recognizing the sequence Ser/Thr-X-Glu/SerP/Asp, where X can be any amino acid residue, and P indicates phosphorylation [25].

Kappeler et al. [5] reported that the most frequent phosphorylation sites in the mature protein were Ser residues in positions 8, 9, 10, 32, 53, 108, 110, and 130. Our findings are consistent with the study of Pauciullo and Erhardt [14], which indicated 12 potential phosphorylation sites in llamas, and with Ryskaliyeva et al. [15], which observed αs2-CN with 7P to 12P in Old-World camels.

Phosphorylation levels in αs2-CN vary significantly among different species. For example, bovine αs2-CN has 18 potential phosphorylation sites, but only 12 serine residues are in the consensus motifs. Nevertheless, αs2-CN with 13P, 14P, and 15P have been found [26]. Until now, only 10 phosphorylation sites of αs2-CN were identified as Ser-X-Glu/SerP motifs [27,28]. Consequently, some threonine residues in bovine αs2-CN must be phosphorylated for the αs2-CN carrying more than 12 phosphates [26]. Similarly, camel αs2-CN-12P needs to be phosphorylated in at least two threonine residues because the serine residues in the consensus motifs are only 10 (Figure 1). The two threonine in positions 118 and 141 fully meet the requirements of the motif Thr-X-Asp/Glu and might be phosphorylated. However, the threonine at position 39, which was predicted to be phosphorylated [15], does not meet the criteria required by the Ser/Thr-X-Glu/SerP/Asp consensus motif and cannot be phosphorylated. 

Furthermore, since phosphorylation is a post-translational modification that occurs after the protein has already reached its tertiary structure, additional potentially phosphorylated sites were identified at positions 114 and 131. These sites follow the motif Glu-X-Thr, which is opposite to the consensus Thr-X-Glu. In a 3D representation of the protein (Supplementary Figure S1), this site might provide the right orientation for the casein kinase to perform phosphorylation. Phosphorylation of threonine 114 was also suggested in llamas [14]. Therefore, the phosphorylation level in camels could potentially reach 14P. 

**Figure 5:**
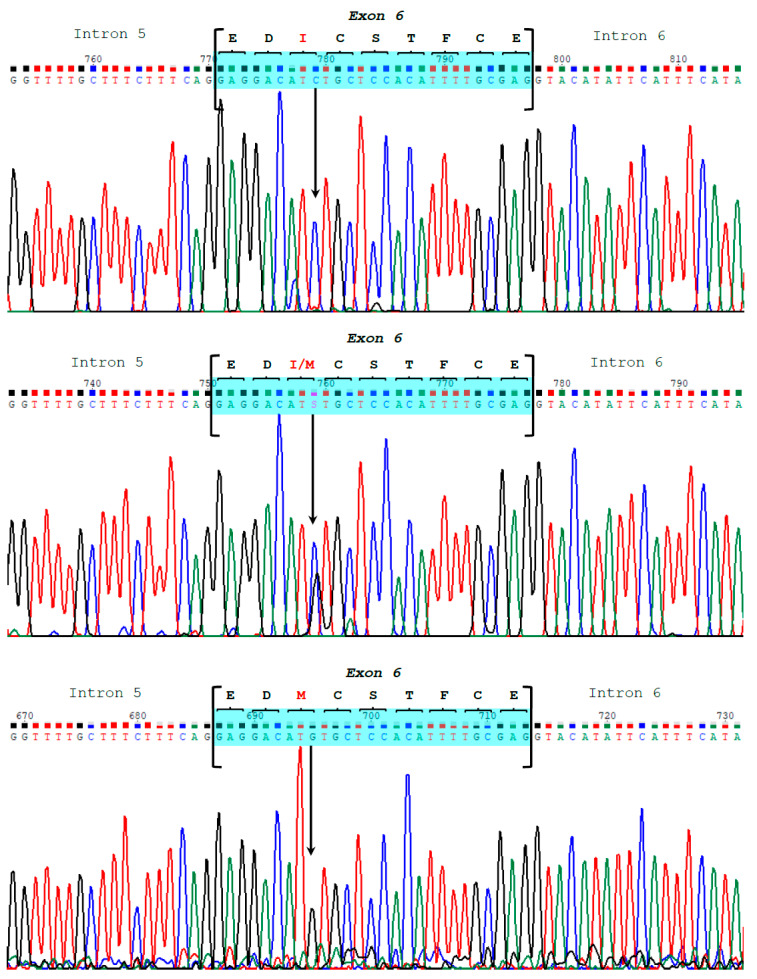
The sequencing chromatograms display the three different genotypes for the SNP g.3639C>G at exon 6 of the *CSN1S2*, which is responsible for the amino acid replacement (p.36Ile>Met) at position 36 of the mature αs2-casein in the Bactrian camel.

Gene sequencing revealed diversity both within and between species. The number of sequenced Bactrian camels for the *CSN1S2* gene was half that of dromedaries, but the former showed slightly more polymorphism (18 SNP) compared to the latter (16 SNP). At the exon level, only two mutations were found (Table 1). In Bactrians, the SNP g.3639C>G at exon 6 resulted in an amino acid change at position 36 of the mature protein (p.36Ile>Met) (Figure 1 and Figure 5). On the other hand, the SNP g.15110G>T at exon 17 of the dromedary *CSN1S2* is located in the 3′-untranslated region. Both mutations represent the first examples of allelic polymorphism in the αs2-casein in Old-World camels.

Previous studies have described two non-allelic αs2-casein variants (αs2-CNsv1 and αs2-CNsv2) in camels [15]. The first isoform results from the skipping of exon 12 (27 bp), which encodes for the nonapeptide ENSKKTVDM. This variant was also described by Kappeler et al. [5]. The second isoform is the outcome of an alternative splicing event due to the identification of a cryptic splice site in intron 16, which partially translated into the decapeptide VKAYQIIPNL [15]. Recently, another splice variant (c.403-9_403-4delTTTTCT) was discovered in Emirates dromedaries, but limited information was reported on this event, and the impact of the variant was considered low [16].

The frequency of the g.3639C>G mutation in the Bactrian population could not be established due to the limited number of available samples. Instead, we genotyped the dromedary population for the SNP g.15110G>T (Figure 2). This mutation occurs in the 3′-UnTranslated Region (UTR), 50 nucleotides downstream of the stop-codon. The 3′-UTR plays a crucial role in the post-transcriptional regulation of gene expression and is particularly significant in suppressing gene expression through microRNA (miRNA) mediation [29]. Thus, variations occurring within these regions could potentially impact binding sites and alter the rate of transcription, mRNA stability, and, consequently, the protein level. 

To investigate whether the SNP g.15110G>T could influence miRNA binding sites, we targeted the 3′UTR of dromedary *CSN1S2* (starting from the first nucleotide of the exon 17). A bioinformatics analysis using the miR database revealed that the mutation affected the binding sites of at least five miRNAs (Table 5). Among these miRNAs, three (miR-298, miR-4418, and miR-3158-5p) showed different target scores, which were consistently higher for the genotype g.15110T/T. Furthermore, the allele T affected the binding of two additional miRNAs (548av-3p and 4662a-3p) that were completely absent for the genotype g.15110G/G. Notably, the mutation g.15110G>T affected the seed sequence (CTATCTT) of the miRNA 4662a-3p. While the TargetScan analysis did not identify milk protein genes as a potential target of this miRNA, it was observed that miRNA 4662a-3p is involved in mammary gland function, as it is significantly upregulated in breast cancer patients [30]. 

The presence of additional putative miRNA target sites may result in a higher downregulation level of αs2-casein for the allele T in the dromedary camel. Conversely, allele G does not appear to cause a potential reduction in αs2 protein synthesis. Additionally, allele G has a lower frequency in the Tunisian dromedary population (Table 3). This suggests that molecular directional selection in favor of this allele could lead to a faster improvement in the αs2-casein yield. 

Comparing the gene sequence in the two species revealed 28 interspecies SNPs (Supplementary Table S2). Twenty-six nucleotide differences were found in the introns, but none of them seemed to affect spliceosome-sensitive sites (donor splice sites GT, branch points, and acceptor splice sites AG). Therefore, intron removal during mRNA maturation is not expected to be disrupted due to decreased sequence affinity for the spliceosome machinery. However, there could be a differential *CSN1S2* expression between Bactrians and dromedaries due to differences in the gene promoter. Specifically, at position 127 of the *CSN1S2* promoter, Bactrians and dromedaries differ in regard to an SNP (T vs. G, respectively) that, in the presence of thymine, creates a new putative consensus binding site for the transcription factor TEC1 (Table 2 and Supplementary Table S2).

TEC1 belongs to the TEA domain (TEAD) family of transcriptional regulators, which control cellular development in many eukaryotes [31]. Although TEAD proteins have interesting functions as genes enhancers, we could not find works in the literature linking them to milk traits. Therefore, our focus was on intraspecific polymorphisms found in the Bactrian *CSN1S2* promoter. Two transitions, G>A, were found (Table 1). The first, which is closer to exon 1, has no impact on the putative consensus sequences for transcription factors (TFs), whereas the second (position 311) created a novel site (position −442/−451) for C/EBP-β (CCAAT/enhancer-binding protein beta; Table 2). 

C/EBP-β belongs to the C/EBP family of transcription factors and plays a central role in regulating gene expression in various tissues, including the mammary gland [32]. In this study, we found four C/EBP motifs in the *CSN1S2* promoter (Table 2). This is not surprising, as C/EBP family elements commonly regulate casein gene expression, even in camels. For example, one C/EBP-α has been identified in the *CSN3* promoter [23], and eight CCAAT/enhancer-binding proteins (α and β) have been detected in *CSN2* [19]. Several motifs have been found in the *CSN1S1* [3], and most of these elements are conserved in other camelids, such as llamas [14]. Hence, it is evident that C/EBP transcription factors play a critical role in the regulation of casein expression, contributing to the production of these proteins in milk, either directly or through their interaction with glucocorticoid (GR) elements [33]. 

The same applies to Octamer transcription factors (Oct-1 and Oct-2), the family of Activator Proteins (AP-1 and AP-4), and the pituitary-specific transcription factor-1 (Pit-1). These three motifs, which are often present in the promoters of camel casein genes, act as enhancers of the gene expression [3,19,23]. Oct-1 is not recognized as a potent transcriptional activator on its own, but it exhibits enhanced activity when combined with other co-activators, such as STAT5A [34] and the TATA box protein (TBP) components [35]. Therefore, it is reasonable to suggest that, as observed for the camel *CSN2* (β-casein), Oct-1 may mediate the stimulation of αs2-casein expression in mammary glands through interaction with these other transcription factors.

Pit-1 belongs to the POU family of transcription factors, which are known for regulating the expression of prolactin and growth hormone [36]. It is also expressed in the mammary gland [37]. The importance of this transcription factor on milk traits has been demonstrated in dairy cattle through a polymorphism found in the Pit-1 gene. Specifically, the A allele has shown significant superiority over the B allele for protein yield and other traits, including milk yield, body depth, angularity, and rear leg set [38]. 

Similarly, AP-1 plays a role in *CSN1S1* (αs-1 casein) gene expression [39] and in glucocorticoid signaling [40] and has been demonstrated to be involved in gene regulation in bovine mammary epithelial cells in response to prolactin [41].

At least two TATA boxes with different binding scores exist (Table 2). The canonical TATA box is located in proximity to the coding region (position −24/−16); it is also conserved in llamas [14] and showed a lower binding score (85.6% vs. 96.4%) than the one farther away (−203/−194). Gene promoters containing multiple TATA boxes have been identified in plants and animals [41,42,43], including an example in the camel β-casein (*CSN2*) gene promoter [19]. While the potential advantage of having multiple TATA binding sites has not been extensively studied, research on luciferase reporter plasmids containing TATA-box mutants of the rat Prolactin-Releasing Peptide gene (PrRP) reveals that the canonical signal 32 bp upstream of the coding region is not required for the initial promoter activity. In contrast, additional deletion of TATA box 92 bp upstream of the coding region almost eliminated PrRP promoter activity, indicating that the more distant TATA box is essential for gene expression [44]. Considering this, a second TATA box with a stronger binding score found in the camel *CSN1S2* might have a similar function that requires further investigation.

Several other putative consensus sequences of TFs have been identified, including STAT5, YY1, and Hfh1 (Table 2). The role of the first two TFs in casein expression is well known. STAT5 is an activator of transcription, often acting in synergy with C/EBPs and GR factors [33,45]. Alternatively, Ying Yang 1 (YY1) serves as a negative regulatory element and mainly participates in repressing casein gene expression [46]. As for Hfh1 (Forkhead homolog-1), its function in the mammary gland is still unknown. However, this transcription factor is related to cell proliferation and differentiation, showing a transient overexpression in the development of the prepuberal rat mammary gland exposed to butyl-benzyl-phthalate (BBP), a plasticizer known to have an endocrine-disrupting action [47].

Caseins represent powerful molecular models for evolutionary studies, as the presence of Interspersed Elements (INEs) within casein genes may hold fundamental clues to understanding species diversification [48]. INEs are member of a larger family of Transposable Elements (TEs) that play a vital role in species diversification and contribute to genomes expansion through various mechanisms of mobilization [49]. Transposable Elements (TEs) are divided into two main categories, referred to as Class I and Class II, based on whether their transposition involves an RNA intermediate or not. Class I TEs, also known as retrotransposons, move within genomes by using a reverse-transcription process that relies on an RNA intermediate derived from a source element. This class can be further categorized into long terminal repeat (LTR) and non-LTR retrotransposons, both with autonomous and nonautonomous mobilization process [49]. Autonomous non-LTR retrotransposons include Long Interspersed Elements (LINEs) such as L1, Bov-B, and L2, while nonautonomous non-LTR retrotransposons are known as Short Interspersed Elements (SINEs), such as Alu, Bov-A2, Bov-tA, tRNA, and MIR, among others [50,51]. Conversely, Class II TEs are DNA transposons that relocate within genomes without involving the reverse transcription of source elements. These are classified into three main subclasses that include Terminal Inverted Repeat (TIR) DNA transposons, such as hATs and mariners; rolling-circle transposons, such as Helitrons; and self-synthesizing DNA transposons, such as Mavericks [49]. INEs have been extensively investigated in ruminants [13,52,53], but studies are also available for camelids *CSN2* [19], *CSN3* [23], and the entire casein cluster [3].

The analysis of *CSN1S2* in dromedary and Bactrian camels allowed us to identify ten repetitive elements (boxes from A to L) in both species (Figure 3). Four LINEs were found in the introns 1, 6, 10, and 11. The first three elements belong to LINEs/L2 (A, B, and H), whereas the repeated element in intron 11 is a LINE/L1 (G), which displayed a polymorphic size (148 bp in Bactrian and 222 bp in dromedary). Additionally, five SINEs were found, two elements in intron 8 (D and E), and the others in introns 9, 14, and 16 (F, I, and L), respectively. The L element (MIRc) also showed size polymorphism (110 bp vs. 163 bp) in Bactrian and dromedary, respectively. Moreover, a DNA transposon was found in intron 7 (hAT-Charlie), measuring 51 bp in length (C). The camel *CSN1S2* gene is also characterized by some low-complexity repeats, all belonging to A-rich regions that, on average, span about 130 bp in both species. Overall, interspersed elements represented the 7.34% and 8.14% of the *CSN1S2* sequence in Bactrian and dromedary, respectively. 

Using the *CSN1S2* gene as a potential marker of evolution [48] and considering that transposition insertions reflect the level of genome-size expansion [54], camels might appear as a relatively young species compared to bovines. However, this is not consistent with information on paleontological records that date in 42–26 Mya the origin of *Camelidae* family [55,56], while *Bovidae* appeared ~23 Mya [57,58]. Camels revealed fewer interspersed elements (10 INEs) than the homologous *Bos taurus CSN1S2* gene (14 INEs) [59]. However, six elements (A, B, F, H, I, and L) are shared among these species and are partially found also in other ruminants [13], indicating a common ancestral origin of these retrotransposons. In contrast, elements C, D, E, and G are specific to camels. Similar findings were observed in the *CSN2* and *CSN3* genes, where dromedary-specific LINEs were identified, distinguishing camels from cattle and suggesting that *Tylopoda* diverged from *Ruminantia* before subsequent transposition events occurred [19,23]. 

The analysis of the *CSN1S2* gene in dromedary and Bactrian camels did not reveal any species-specific retro-transposition elements. This result was expected, as nearly 95% of repetitive DNA in the casein cluster is shared among two or more camelid species, with only the remaining 5% being specific to a particular species [3]. However, it is interesting to note that four elements (C, D, E, and G) might indicate their presence in the *CSN1S2* gene of an ancient camel genome before dromedaries and Bactrians diverged further. 

Retrotransposons are considered significant drivers of genome evolution due to their influence on genome stability through various mechanisms, including facilitating exonization, regulating epigenetic modifications, impacting RNA editing, generating microsatellites, and introducing mutations [60]. For instance, in the river buffalo *CSN1S2,* transposable elements showed an almost double incidence of genetic variation (SNP, insertion/deletion) compared to the rest of the gene, suggesting that they play a major role in generating genetic variability [13]. 

In contrast, the impact of transposable elements on the level of genetic diversity in camel *CSN1S2* is much less severe. Only a few SNPs (4 out of 18 in Bactrian and 2 out of 16 in dromedary) were found in the retrotransposons, contributing to 17.6% of the total genetic diversity found. This result reflects a lower level of genetic diversity found in the casein genes of camelids [3,14,18,19,20,22,23,61,62] compared to ruminants [13,63,64,65,66]. These findings are consistent with the observations made by other authors, indicating a significant reduction in genetic diversity due to at least two bottlenecks during the history of camel domestication [56,67,68].

## 5. Conclusions

A casein gene study and the identification of its genetic diversity are highly interesting due to the potential practical applications of variants that positively impact production in the dairy industry. The current study represents the first complete analysis of *CSN1S2* in Bactrian and dromedary camels, providing valuable insights into the gene characterization. Through a comprehensive molecular analysis, we elucidated the genetic structure, identified the genetic diversity and the first alleles in both species, described the integration of retrotransposons, and discovered interesting elements of gene regulation in the promoter and 3′ UTR.

In the dromedary, the SNP g.15110G>T shows promise to functionally impact miRNA 4662a-3p. The low frequency of the g.15110G allele offers an opportunity for genetic improvement through molecular-assisted selection. Similarly, in the Bactrian, the SNP g.311G>A creates a novel site for C/EBP-β in the gene promoter, and the SNP g.3639C>G is responsible for the first amino acid variant (p.36Ile>Met) described so far. All of these polymorphisms will be useful for association studies with milk protein yield, opening the door to future investigations in this relatively little explored research area for these species.

## Figures and Tables

**Table 1:** Intraspecies polymorphisms detected by sequencing the *CSN1S2* gene in *C. bactrianus* and *C. dromedarius*. The corresponding mutations detected in our investigated samples, indicated as gray cells, are R = A/G, Y = C/T, W = A/T, K = G/T, M = A/C, and S = G/C. Numbering refers to GenBank IDs OQ730238 and OQ730239. For a comparative analysis, the position of each polymorphism is also indicated in the other species, as well as the corresponding nucleotide.

Position	Nucleotide	Bactrian Present Study (OQ730238)	Bactrian Genome (NW_011517196)	Nucleotide	Dromedary Present Study (OQ730239)	Dromedary Genome (NW_011591251)
Promoter	311	R	G	311	G	G
	674	R	G	674	G	G
Intron 1	845	Y	T	845	T	T
	865	W	T	865	T	T
	905	Y	C	905	C	C
	1086	R	A	1086	A	A
	1197	R	G	1197	G	G
	1294	R	G	1294	G	G
	1399	R	G	1399	G	G
	1568	T	T	1568	K	T
Intron 3	2848	R	A	2857	A	A
	2854	G	G	2863	R	G
Intron 5	3530	Y	T	3539	T	T
	3587	W	A	3596	A	A
Exon 6	3639	S	C	3648	C	C
Intron 6	3757	G	G	3766	R	G
	4429	C	C	4438	Y	C
Intron 7	5412	R	G	5421	G	G
	5798	G	G	5807	K	G
Intron 8	6028	A	A	6037	R	A
	6281	G	G	6290	R	G
	7198	A	A	7236	M	A
Intron 9	7942	G	G	7980	K	G
	8139	S	C	8177	C	C
	8169	W	T	8207	T	T
Intron 10	8623	C	T	8659	Y	T
	8767	A	A	8793	R	A
Intron 11	9662	T	T	9698	Y	T
Intron 12	9883	T	T	9919	W	T
Intron 14	11329	A	A	11368	M	A
	12073	Y	C	12112	C	C
Intron 15	12261	A	A	12300	W	A
Intron 16	13842	Y	T	13881	T	T
Exon 17	15069	T	T	15110	K	T
Total		18			16	

**Table 2:** Transcription factors and consensus motifs detected in the 5′-flanking regions of Old-World camels. Sense strand (5’ to 3’) is indicated with +, whereas the complementary strand (3’ to 5’) with −. The negative numbering identifies the nucleotide distance from the first nucleotide of exon 1. * Additional consensus motif generated by the SNP g.305 G>A in the presence of the adenine (underlined).

Transcription Factor	Consensus Motif	Signal Sequence	Strand	Score	*C. bactrianus*	*C. dromedarius*
AP-1	NNTGACTCANN	CCTGACTCCCT	+	0.913	−677/−667	−677/−667
TEC1	TNCATTCYWW	TTCATTCCAT	+	0.985	−620/−629	-
AP-4	NNCAGCTGNN	CACAGCTGGT	+	0.989	−591/−582	−591/−582
Oct-1	CWNAWTKWSATRYN	CACAATTAAATATG	+	0.946	−573/−560	−573/−560
Pit-1a	NNGAATATKCANNNN	AATATGAATATTATT	−	0.944	−565/−551	−565/−551
C/EBP-β	RNRTKNNGMAAKNN	AAGTTAAGAAAGTA	+	0.908	−527/−514	−527/−514
AP-4	NNCAGCTGNN	GAGAGCTGAG	−	0.934	−482/−473	−482/−473
C/EBP-β *	RNRTKNNGMAAKNN	GACTTGCATAAGACT	−	0.909	−453/−439	-
YY1	CCATNTWNNNW	CCATATTTTTA	+	0.899	−436/−426	−436/−426
Pit-1a	TGAATAWNWA	TGAATATGAA	+	0.859	−404/−395	−404/−395
Oct-1	CWNAWTKWSATRYN	AATATGAAAAATGT	−	0.847	−402/−389	−402/−389
Oct-1	NNNRTAATNANNN	GTATTAATGAAAT	+	0.870	−378/−366	−378/−366
Oct-1	CWNAWTKWSATRYN	CACATCCAAAATAT	−	0.890	−356/−343	−356/−343
C/EBP-α	NNTKTGGWNANNN	TATTTGTTTAAAG	+	0.901	−333/−321	−333/−321
STAT5A	TTCCCRKAA	TTCTAGGAA	−	0.956	−290/−282	−290/−282
Hfh1	NAWTGTTTATWT	AAAAAAAAAATC	−	0.924	−283/−272	−283/−272
C/EBP-α	TRRCCAATSRN	GAACCACACAG	+	0.799	−269/−259	−269/−259
HNF-3/FOXA1	NNNTRTTTRYTY	CTATAAATAATT	−	0.861	−222/−211	−222/−211
GR	NTGCGTRGGCGK	ATTCCTACACAC	+	0.787	−213/−202	−213/−202
TATA box	NCTATAAAAR	ACTATAAAAT	+	0.964	−203/−194	−203/−194
STATx	TTCCCRKAA	TTCTTATAA	+	0.905	−187/−179	−187/−179
STAT5A	TTCCCRKAA	TCCTTGGAA	+	0.813	−147/−139	−147/−139
STAT5A	TTCCCRKAA	TTCTTAGAA	+	0.912	−91/−83	−91/−83
TATA box	WTATAAAW	ATTTAAAT	+	0.856	−24/−16	−24/−16
		Total elements			24	22

**Table 3:** Genotyping data and allele frequency of the SNP g.15110G>T at the *CSN1S2* gene in Tunisian *C. dromedarius.*

	Genotype Distribution				Allelic Frequency	
	TT	GT	GG	Total	T	G
*Observed*	83	61	13	157	0.723	0.277
*Expected*	82.05	62.89	12.05			

χ^2^ = 0.142.

**Table 4:** Interspersed elements discovered in the *CSN1S2* gene of the Old-World camels and their position in the sequence. Sense strand (5’ to 3’) is indicated with +, whereas the complementary strand (3’ to 5’) with −. Comparison with the homologous *Bos taurus* gene available in NBCI under the following ID number: M94327.1.

	*Camelus bactrianus CSN1S2* GenBank ID: OQ730238				*Camelus dromedarius CSN1S2* GenBank ID: OQ730239				*Bos taurus CSN1S2* GenBank ID: M94327.1			
	Position		Name	Strand	Position		Name	Strand	Position		Name	Strand
	Intron	Nucleotide			Intron	Nucleotide			Intron	Nucleotide		
LINEs												
LINE 1												
	10	8635/8784	G:L1MA10	+	10	8613/8834	G:L1MA10	+				
LINE 2												
	1	1270/1320	A: L2a	+	1	1270/1319	A: L2a	+	1	3953/4000	A:L2a	+
	6	4198/4270	B:L2a	−	6	4207/4279	B:L2a	−	6	8069/8151	B:L2a	−
	11	9528/9624	H:L2a	−	11	9564/9659	H:L2a	−	12	12212/12306	H:L2a	−
DNA elements												
hAT-Charlie	7	5319/5370	C:MER5A	+	7	5328/5379	C:MER5A	+				
SINEs												
MIRs												
	8	6562/6785	D:MIRb	+	8	6572/6794	D:MIRb	+				
	8	7330/7385	E:MIRb	−	8	7368/7423	E:MIRb	−				
	9	8093/8232	F:MIRc	−	9	8131/8270	F:MIRc	−	11	10742/10888	F:MIRc	−
	14	11593/11775	I:MIRb	−	14	11632/11814	I:MIRb	−	15	14773/15100	I:MIRb	−
	16	13828/13938	L:MIRc	−	16	13867/14030	L:MIRc	−	17	19404/19481	L:MIRc	−
Alu/B1									2	4894/5086	M:BMF	+
									8	9534/9772	N:ARS2	+
									12	11563/11967	O:BDF	+
									13	13489/13703	P:BMF	+
									15	14664/14947	Q:BDF	+
									17	16405/16608	R:BMF	+
									17	17300/17827	S:ARS2	−
									17	19128/19403	T:BMF	+

**Table 5:** Putative mature sequences for microRNAs found in the Old-World camel’s *CSN1S2* gene, using the miR database and *Homo sapiens* as the reference species. Underlined nucleotide in the target sequence of miR-4662a-3p is the mutated nucleotide for the SNP g.15110G>T.

miRNA Name	miRNA Sequence	Seed Location	Custom Target Sequence	Target Score	
				T/T	G/G
hsa-miR-298	AGCAGAAGCAGGGAGGUUCUCCCA	20	CTTCTGCA	93	87
hsa-miR-4418	CACUGCAGGACUCAGCAG	23	CTGCAGT	83	76
hsa-miR-3158-5p	CCUGCAGAGAGGAAGCCCUUC	22	TCTGCAG	81	65
hsa-miR-548av-3p	AAAACUGCAGUUACUUUUGC	25	GCAGTTT	66	-
hsa-miR-4662a-3p	AAAGAUAGACAAUUGGCUAAAU	16	CTATCTT	52	-

## Data Availability

The data presented in this study are available upon request from the corresponding author.

## Supplementary Materials

The following supporting information can be downloaded at https://www.mdpi.com/article/10.3390/ani13172805/s1. Figure S1: The 3D representation of the amino acids 123-137 of αs2-casein is shown in both the normal primary frame (A) and the opposite frame (B), providing the right orientation (Thr-X-Glu) for the casein kinase to perform phosphorylation; Table S1: Amplicons, sequences and annealing temperatures of the primers used for the characterization of *CSN1S2* gene in the Old-World camels (*C. bactrianus and C. dromedarius*); Table S2: Inter-species polymorphisms detected by sequencing the *CSN1S2* gene in *C. bactrianus* and *C. dromedarius*; Figure 2 Gel electrophoresis original figure.
