# Supplementary material for: Sequencing and Characterization of αs2-Casein Gene (CSN1S2) in the Old-World Camels Have Proven Genetic Variations Useful for the Understanding of Species Diversification
Source: Animals (Basel). 2023 Sep 4;13(17):2805. doi: 10.3390/ani13172805 (PMC10487017; doi:10.3390/ani13172805)
Supplement: Supplementary file 1 [file animals-13-02805-s001.zip › Figure S1.pdf]

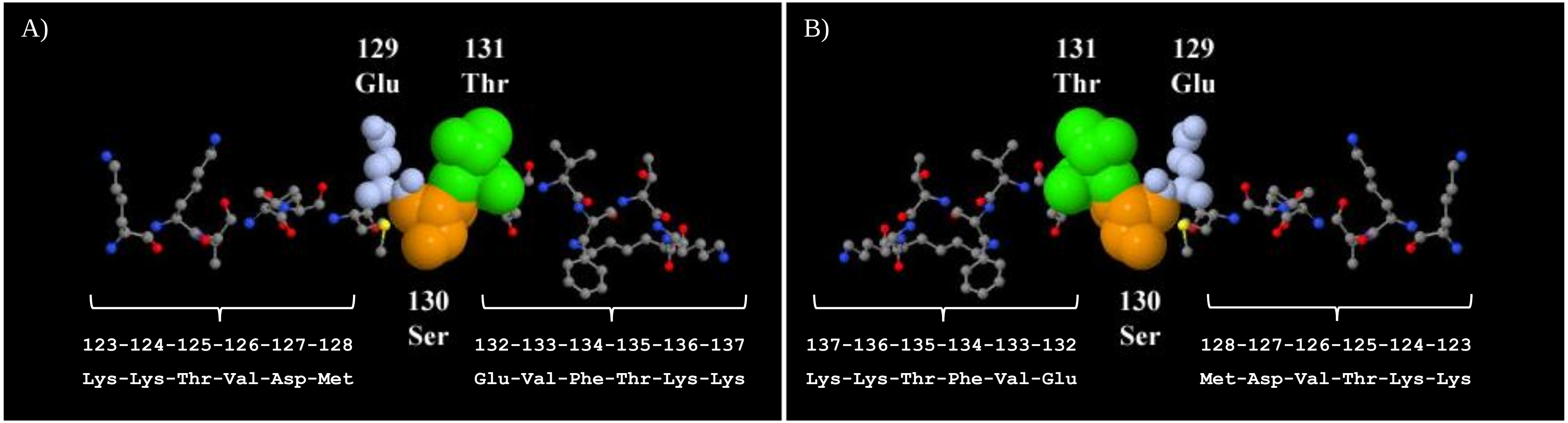

Figure S1. The 3D representation of the amino acids 123-137 of αs2-casein is shown in both the normal primary frame (A) and the opposite frame (B), providing the right orientation (Thr-X-Glu) for the casein kinase to perform phosphorylation.
