# Supplementary material for: Sequencing and Characterization of αs2-Casein Gene (CSN1S2) in the Old-World Camels Have Proven Genetic Variations Useful for the Understanding of Species Diversification
Source: Animals (Basel). 2023 Sep 4;13(17):2805. doi: 10.3390/ani13172805 (PMC10487017; doi:10.3390/ani13172805)
Supplement: Supplementary file 1 [file animals-13-02805-s001.zip › Table S1.pdf]

Amplicon	Primers	Sequence	Tm °C	Size (bp)
1	*Cf CSN1S2 Prom F4*	5’-ATAACTGTCAGTGATTTCTC-3’	53.6	791
	Cf CSN12 Ex 1 R	5’- TGAAGGGAAGACAAGTA-3’		
2	*Cd CSN1S2 Prom F*	5’- CACACAAACACAAACTATAAATAA-3’	54.1	925
	Cd CSN1S2 Int 1 R	5’- ACATAATGTAAAATCTTGGTTTC-3’		
3	*Cd CSN1S2 Int 1 F*	5’- TTTCATTACTGTATCTTCACG-3’	53.9	1194
	Cd CSN1S2 Int 3 R1	5’- AATTTCTCTGACTCTGTAC-3’		
4	*Cd CSN1S2 Int 2 F*	5’- AAACAGAATTCTAAAAGCCC-3’	55.3	995
	Cd CSN1S2 Ex 4 R	5’- CTGAGAGACGTTGATAGATT-3’		
5	*Cd CSN1S2 Int 3 F*	5’- ATATTTTTCCTCTCTTCTCTG-3’	54.7	1033
	Cd CSN1S2 Int 6 R	5’- AGAATCTGGGAATCAAATATAT-3’		
6	*Cd CSN1S2 Ex 6 F*	5’- GACATCTGCTCCACATTT-3’	53.4	1340
	Cd CSN1S2 Int 7 R	5’- TATATAATCAAAATCTGGTGA-3’		
7	*Cd CSN1S2 Int 7 F1*	5’- CCTGGTTACATTGCTTTTAT- 3’	54.3	1166
	Cd CSN1S2 Ex 8 R	5’- ACTTCAGCTGATTCCTTA-3’		
8	*Cd CSN1S2 Int 7 F*	5’-CTGTAAGGAACATAAAGGAAG -3’	57.7	1897
	Cd CSN1S2 Int 8 R1	5’- CAGAACCAAATTACTTAGACCT-3’		
9	*Cd CSN1S2 Int 8 F*	5’- TTAAAGGTCTAAGTAATTTGGT-3’	53.8	869
	Cd CSN1S2 Int 10 R	5’- TTATTGCTTACAAAACTAGATC-3’		
10	*Cd CSN1S2 Ex 10 F*	5’- ATACAGAACACAGAGCAG-3’	54.8	1587
	Cd CSN1S2 Int 12 R	5’- TAGTATGAGAGATGAAAAAGAA-3’		
11	*Cd CSN1S2 Ex 12 F*	5’- GGAAAATTCAAAAAAGACTGTT-3’	53.9	1490
	Cd CSN1S2 Ex 14 R	5’- TTCTTCAGTCAATTCAGTTTTC-3’		
12	*Cd CSN1S2 Ex 12 F*	5’- GGAAAATTCAAAAAAGACTGTT-3’	52.6	2405
	Cd CSN1S2 Ex 15 R	5’- AGAGGAATGTCTGATAATAT-3’		
13	*Cd CSN1S2 Ex 15 F*	5’- ATCTCAAGACTGTTTATCAATA-3’	54.1	885
	Cd CSN1S2 Ex 16 R	5’- CATAATCAAGTAGAAGCAGT-3’		
14	*Cd CSN1S2 Int 15 F*	5’- AGCTTTTGATTTTGACAAGTCACTT-3’	56.05	2057
	Cd CSN1S2 Int 16 R1	5’- GCATACCAAAGTAATATTTATA-3’		
15	*Cd CSN1S2 Int 16 F*	5’- GGATAATTAAATGTTCCTTCAAAA-3’	54.3	767
	Cd CSN1S2 3-End R	5’- GTGAGAAGTAAAACTGAAGT-3’		

Table S1. Amplicons, sequences and annealing temperatures of the primers used for the characterization of *CSN1S2* gene in the Old World camels (*C. bactrianus* and *C. dromedarius*).
