# Supplementary material for: Sequencing and Characterization of αs2-Casein Gene (CSN1S2) in the Old-World Camels Have Proven Genetic Variations Useful for the Understanding of Species Diversification
Source: Animals (Basel). 2023 Sep 4;13(17):2805. doi: 10.3390/ani13172805 (PMC10487017; doi:10.3390/ani13172805)
Supplement: Supplementary file 1 [file animals-13-02805-s001.zip › Table S2.pdf]

Position	Nucleotide	Bactrian present study (OQ730238)	Nucleotide	Dromedary present study (OQ730239)
Promoter	37	T	37	C
	127	T	127	G
Intron 1	1339	T	1339	A
	1628	C	1628	T
Intron 2	1766	C	1766	A
	1862	C	1862	T
	2134	G	2134	C
Intron 3	2393	A	2401	G
	2530	C	2538	T
Intron 5	3510	T	3519	C
Intron 6	3853	T	3862	C
	4120	C	4129	T
	4447	T	4456	C
Intron 7	5166	G	5175	C
	5773	G	5782	A
Intron 8	6644	T	6653	C
	7039	A	7048	C
	7211	C	7249	T
Intron 9	8092	G	8130	T
Intron 10	8916	T	8952	G
Intron 12	10165	T	10201	A
	10552	G	10588	A
Intron 14	11364	G	11403	A
	11660	C	11699	A
Intron 16	13140	G	13179	A
	13990	A	14029	C
	14350	C	14389	A
	14681	G	14722	A
**Total**		**28**		**28**

Table S2. Inter-species polymorphisms detected by sequencing the *CSN1S2* gene in *C. bactrianus* and *C. dromedarius*.
