# Supplementary figures and images for: Sequencing and Characterization of αs2-Casein Gene (CSN1S2) in the Old-World Camels Have Proven Genetic Variations Useful for the Understanding of Species Diversification
Source: Animals (Basel). 2023 Sep 4;13(17):2805. doi: 10.3390/ani13172805 (PMC10487017; doi:10.3390/ani13172805)

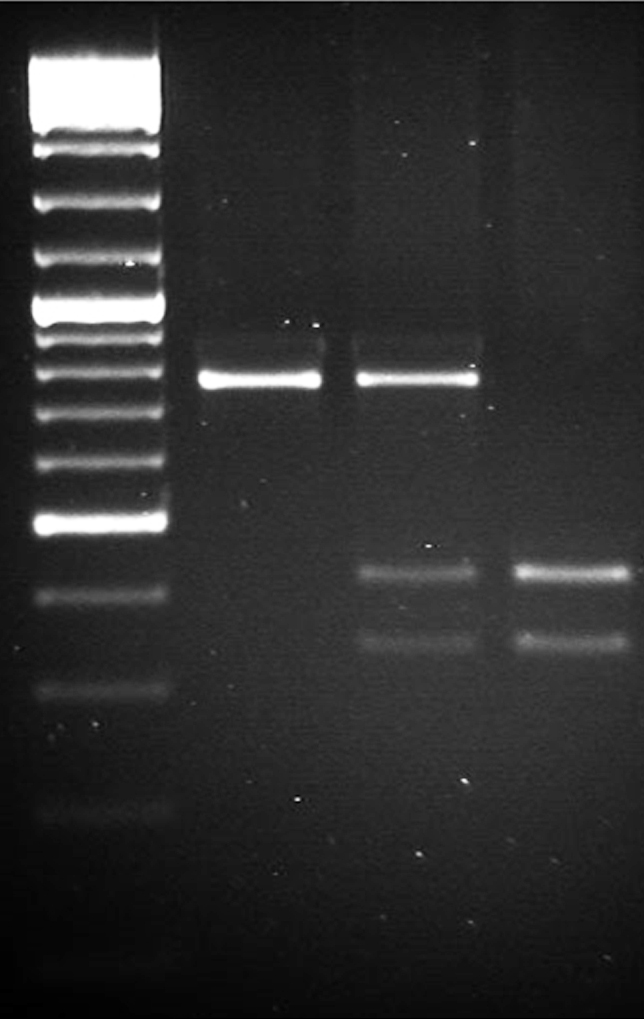

Supplement: Supplementary file 1 [file animals-13-02805-s001.zip › Figure 2 original.jpg]
